# Postnatal Mouse Brain Region‐Resolved Peptidomic Resource of Conditional Ndel1 Loss: Comparison of Acidic and Alcoholic Extraction Strategies

**DOI:** 10.1111/jnc.70330

**Published:** 2025-12-23

**Authors:** João V. Nani, Jackelinne Yuka Hayashi, Joana D. Campeiro, Guilherme Araújo Câmara, Atsushi Saito, William Y. Oyadomari, Akira Sawa, Atsushi Kamiya, Alexandre K. Tashima, Mirian A. F. Hayashi

**Affiliations:** ^1^ Department of Pharmacology, Escola Paulista de Medicina (EPM) Universidade Federal de São Paulo (UNIFESP) São Paulo SP Brazil; ^2^ Department of Biochemistry, Escola Paulista de Medicina (EPM) Universidade Federal de São Paulo (UNIFESP) São Paulo SP Brazil; ^3^ Department of Psychiatry and Behavioral Sciences Johns Hopkins University School of Medicine Baltimore Maryland USA; ^4^ Department of Neuroscience Johns Hopkins University School of Medicine Baltimore Maryland USA; ^5^ Department of Biomedical Engineering Johns Hopkins University School of Medicine Baltimore Maryland USA; ^6^ Department of Genetic Medicine Johns Hopkins University School of Medicine Baltimore Maryland USA; ^7^ Department of Pharmacology Johns Hopkins University School of Medicine Baltimore Maryland USA; ^8^ Department of Mental Health Johns Hopkins University Bloomberg School of Public Health Baltimore Maryland USA

**Keywords:** brain protein expression, conditional knockout, cytoskeletal proteins, mass spectrometry, Ndel1, peptidome, protein–protein interaction, proteostasis

## Abstract

The oligopeptidase Ndel1 (NudE neurodevelopment protein 1 like 1) is a multifunctional protein implicated in neurodevelopmental processes, intensively investigated as a potential biomarker in psychiatric disorders. While its roles in regulating the cytoskeleton are well‐studied, the global consequences of its loss on the brain's peptide landscape are unknown. This study presents a comprehensive, region‐resolved peptidomic resource detailing the consequences of postnatal Ndel1 loss in the mouse cortex, hippocampus, striatum, and cerebellum. We also validated this method of microwave protease inactivation followed by acidic and organic extractions by means of this peptidome analysis across several brain regions. More specifically, using a conditional knockout mouse model with Ndel1 deletion in forebrain excitatory neurons, we employed complementary acidic and alcoholic extraction workflows coupled to liquid chromatography–tandem mass spectrometry (LC–MS/MS). We generated a comparative atlas of differentially abundant peptides (DAPs), identifying hundreds of peptide changes across the different brain regions and extraction methods. Gene Ontology analysis of the inferred source proteins revealed alterations in pathways related to cytoskeletal organization, synaptic function, and cellular metabolism. This dataset provides a foundational resource for generating new hypotheses about Ndel1's region‐specific functions and serves as a valuable reference for the neurodevelopmental and neuropeptidomics communities. Understanding these Ndel1‐driven changes is crucial, providing valuable insights into the pathophysiology of neurodevelopmental and mental disorders linked to reduced Ndel1 activity, such as schizophrenia, autism, and bipolar disorder. This holistic view may reveal novel therapeutic targets for these complex conditions.

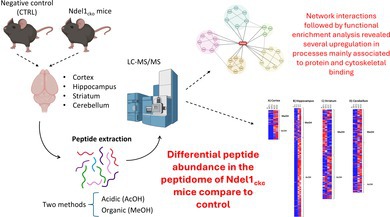

## Graphical abstract

The Ndel1 protein is crucial for neurodevelopment, but its role in the brain peptidome is unknown. Here we compared the peptidome of Ndel1 conditional knockout (Ndel1_cKO) mice with controls (CTRL) in four brain regions. Peptides were extracted using acidic (Acetic Acid, AcOH) and organic (Methanol, MeOH) methods and analyzed by Liquid Chromatography–Tandem Mass Spectrometry (LC–MS/MS). We found differential peptide abundance in Ndel1_cKO mice, revealing widespread dysregulation in pathways associated with cytoskeletal organization and protein binding. This resource provides new insights into Ndel1's function and its role in neurodevelopmental disorders.

**Abbreviations**
ACUC: Animal Care and Use Committee
ASD: autism spectrum disorder
BD: bipolar disorder
BP: biological process
CC: cellular component
cKO: conditional knockout
CMV: cytomegalovirus
DAPs: differentially abundant peptides
DISC1: *Disrupted‐in‐Schizophrenia 1*
FEP: first‐episode psychosis
GO: Gene Ontology
LC–MS/MS: liquid chromatography–tandem mass spectrometry
MF: molecular function
Ndel1: NudE neurodevelopment protein 1 like 1
P3: postnatal Day 3
PCA: principal component analysis
POP: prolyl oligopeptidase
PPI: protein–protein interaction
RRID: research resource identifier
SZ: schizophrenia

## 1 Introduction

The NudE neurodevelopment protein 1 like 1 (Ndel1) oligopeptidase is an enzyme with potential as a biomarker for supporting the diagnosis of schizophrenia (SZ) (Gadelha et al. 2013) and other mental disorders including bipolar disorder (BD) (Dal Mas, Carvalho, et al. 2019), as well as proven to be useful for monitoring the responses to pharmacotherapy in early‐stage SZ (Dal Mas, Nani, et al. 2019; Nani et al. 2024) and in reversion of zika virus induced microcephaly (Christoff et al. 2023). More recently, a growing interest in Ndel1's potential as a neurodevelopmental biomarker was motivated by the demonstration of the association of this oligopeptidase activity with aberrant neurodevelopment pathologies, such as in idiopathic autism spectrum disorder (ASD) showing macrocephaly associated with severe clinical symptoms (Nani, Fonseca, et al. 2020; Courchesne et al. 2024).

As an oligopeptidase, Ndel1 specifically hydrolyzes small substrates, typically peptides of 8–13 amino acid residues, while being unable to hydrolyze larger proteins or peptides exceeding 30 residues (Hayashi et al. 1996; Hayashi et al. 2005). The role of Ndel1 oligopeptidase in neurite outgrowth, neuronal migration, and embryonic brain formation is well‐established (Youn et al. 2009; Hayashi et al. 2010; Hippenmeyer et al. 2010; Rodríguez et al. 2020; Nani, Fonseca, et al. 2020; Courchesne et al. 2024). However, how its specific oligopeptidase activity contributes to brain development and neuronal function remains largely unresolved. Exploration of Ndel's in vivo roles has been limited because current inhibitors, such as polyclonal antibodies and small chemical compounds, are unsuitable for in vivo applications (Nani et al. 2023). Consequently, the potential in vivo roles of Ndel1 oligopeptidase activity remains largely unexplored.

Ndel1 interaction with other key cytoskeletal proteins, most notably with the DISC1 (*Disrupted‐in‐Schizophrenia 1*) protein, has been extensively characterized by many groups, revealing that DISC1 protein competitively inhibits Ndel1 oligopeptidase activity, also modulating the neurite outgrowth (Hayashi et al. 2005; Kamiya et al. 2006; Hayashi et al. 2010; Habibi et al. 2023). DISC1 is a crucial scaffolding protein in the brain that regulates several key processes, and knockdown during development leads to behavioral abnormalities later in life, while DISC1 overexpression leads to impaired brain formation (Nani, Fonseca, et al. 2020). Given that over 95% of Ndel1 is complexed to DISC1 in the rat brain (Hayashi et al. 2005), this strong interaction has motivated studies linking Ndel1 oligopeptidase activity to psychiatric and neurological disorders, including SZ, BD, first‐episode psychosis (FEP), and ASD (Gadelha et al. 2013; Dal Mas, Carvalho, et al. 2019; Dal Mas, Nani, et al. 2019; Rodríguez et al. 2020; Nani et al. 2022; Nani et al. 2023; Courchesne et al. 2024). More recently, lower Ndel1 activity has also been associated with microcephaly induced by Zika virus infection during pregnancy (Christoff et al. 2023). Notably, the link to microcephaly is particularly compelling, as this condition is often recognized as a cell‐cycle disorder resulting from neuronal progenitor cell‐cycle arrests (Doobin et al. 2017), and the Ndel1 expression modulation was also confirmed by RNA‐seq (Nani et al. 2025).

Since constitutive loss of Ndel1 results in neuronal migration defects and early embryonic lethality (Sasaki et al. 2005; Nani, Fonseca, et al. 2020), the creation of a conditional knock‐out (cko) to probe the roles of Ndel1 in postnatal brains was crucial to demonstrate its roles in hippocampus integrity independent of its function in CA1 pyramidal neuron migration (Jiang et al. 2016), but mediated by microtubule fragmentation (Kiroski et al. 2020), as these animals exhibit fragmented microtubules, synaptic pathologies, and dendritic dispersion (Jiang et al. 2016). The long‐lasting upregulation of Ndel1 expression within the hippocampus following a pilocarpine‐evoked repetitive seizure paradigm also reinforces Ndel1's importance for hippocampus function and integrity (Choi et al. 2016; Zhu et al. 2020). The coordinated activity of Ndel1 with LIS1 and dynactin/cargo‐adapters is also well documented (Garrott et al. 2022; Mun et al. 2023; Okada et al. 2023), particularly in regulating axonal mitochondrial trafficking in mature neurons (Shao et al. 2013; Pandey et al. 2022). However, none of these studies evaluate widely the protein expression modulation in response to loss of Ndel1 expression.

Thus, clarifying the direct consequences of the full‐length non‐mutated Ndel1 protein loss in vivo is crucial for uncovering the molecular pathways associated with this neurodevelopmental biomarker. In this context, the primary aim of this study was to elucidate how reduced Ndel1 expression affects the brain's proteome, which was performed by a peptidome analysis across several brain regions following microwave inactivation of proteases followed by acidic and organic extractions of brains from Ndel1 conditional knockout mice (Ndel1_cko_), based on a cre/loxp system essentially following established procedures reported by others (Sasaki et al. 2005). We hypothesize that a comprehensive peptidome analysis in Ndel1_cko_ mice would provide a valuable atlas of molecular changes, generating a resource for future hypothesis‐driven studies into Ndel1‐related dysfunctions. These findings could serve as a foundation for developing targeted therapeutic strategies to address the detrimental consequences of Ndel1 deficiency, which was reported in diverse pathological conditions, including neurodevelopmental pathologies such as microcephaly, macrocephaly, among others, as well as in mental disorders, such as SZ, BD, FEP, ASD, among others. In addition, this study allowed us to validate and compare the acidic and organic extraction of different brain regions of postnatal mice.

## 2 Animals

The conditional knockout mice (Ndel1_cko_) were generated essentially as previously described (Sasaki et al. 2005). The mice were provided by Dr. Shinji Hirotsune (Osaka City University School of Medicine, Osaka, Japan) in collaboration with Dr. Anthony Wynshaw‐Boris (Case Western Reserve University School of Medicine, Ohio, USA). Ndel1_cko_ mice homozygous for a loxP‐flanked *Ndel1* allele (*Ndel1*flox/flox; JAX Strain #: 026958; RRID:IMSR_JAX: 026958) were crossed with a transgenic line expressing Cre‐recombinase under the control of the human cytomegalovirus (CMV) promoter (e.g., B6.C‐Tg(CMV‐cre)1Cgn/J; JAX Strain #:006054; RRID:IMSR_JAX:006054). The CMV promoter typically drives Cre‐recombinase expression early in embryogenesis, leading to the deletion of loxP‐flanked gene segments in most tissues. However, complete homozygous loss of the *Ndel1* gene is known to result in early embryonic lethality. The survival of homozygous knockout pups to postnatal Day 3 (P3) in this study indicates that the Cre‐mediated recombination was likely incomplete or mosaic. This pattern of mosaic expression, a phenomenon that can occur with some ubiquitous Cre drivers, would allow enough cells to retain *Ndel1* function, thereby enabling the animals to bypass the otherwise lethal phenotype.

The animals were backcrossed with C57B6/J mice (Jackson Laboratory, Strain #: 000664) for at least seven generations. During backcrossing, the presence of the loxP‐flanked Ndel1 allele was confirmed by PCR of genomic DNA from the tail. During backcrossing, the presence of the loxP‐flanked Ndel1 allele was confirmed by PCR of genomic DNA from the tail by using specific primers (forward: 5′‐TGTCTGCAAAGACTAACTAGGCG‐3′, reverse: 5′‐TCGTATAATGTATGCTATACGAAGTTATCC‐3′). The loxP‐flanked allele homozygosity and heterozygosity were distinguished by PCR with specific primers (forward: 5′‐TGTCTGCAAAGACTAACTAGGCG‐3′, reverse: 5′‐TTGGAGAATGACTTCACTTCAGT‐3′). After cross‐mating with Cre reporter lines, Ndel1 allele deletion was detected by PCR with specific primers (forward: 5′‐TGTCTGCAAAGACTAACTAGGCG‐3′, reverse: 5′‐TGCAGGCACCTGCACATAAGTGG‐3′) (Sasaki et al. 2005).

Male pups were used for all experiments, and tissues were collected at P3. Animals were housed in standard polycarbonate cages (2–5 mice per cage) with their dam under a 12‐h light/dark cycle with controlled temperature and humidity. Animals had *ad libitum* access to standard rodent chow and water. All animal procedures were performed in accordance with NIH guidelines and with approval from the Johns Hopkins University Institutional Animal Care and Use Committee (ACUC) under the code MO23M218.

## 3 Brain Collection and Peptide Extraction

We analyzed the effect of Cre‐mediated Ndel1 inactivation on protein expression by comparing the brain proteomes of two groups of mice. The experimental group consisted of Ndel1 conditional knockouts (*n* = 2), which were compared to littermate controls lacking Cre‐recombinase (*n* = 2), at age postnatal Day 3 (P3). Neonatal pups were first anesthetized via hypothermia by being placed on crushed ice for 2–4 min. They were then euthanized by decapitation, and their brains were immediately extracted and separated into left and right hemispheres and subjected to focused microwave irradiation (8 s at full power) to instantly inactivate endogenous proteases and to preserve the in vivo peptidome (Che et al. 2007). Regions of interest (cortex, hippocampus, striatum, and cerebellum) were separated and stored immediately in liquid nitrogen. Then, tubes containing these tissue samples were stored at −80°C until use.

For aqueous extraction, tissue samples (*n* = 16 independent samples) were homogenized in a cold extraction solution (0.25% acetic acid, 5 μL/mg tissue) using sonication (3 cycles of 8 s each, 70% amplification) and centrifuged at 15000×*g* for 45 min at 4°C. The supernatant containing proteins and peptides was transferred to a filter (Microcon YM‐10, Millipore, Bedford, MA, USA; Cat. #UFC501096) with a 10 000 Da molecular weight cut‐off and centrifuged at 14000×*g* for 45 min at 4°C. The peptide‐containing filtrate was immediately frozen and stored at −80°C for further analysis. The pellet remaining after the centrifugation of the acidic homogenate was used for the subsequent organic extraction step.

For organic extraction, 3 μL of cold 0.25% acetic acid and 0.75 μL of methanol per mg of tissue (20% methanol extraction) were added to the recovered precipitate (*n* = 16 independent samples), followed by homogenization and centrifugation as described above. The supernatant was collected and kept on ice. Then, 1.87 μL of cold 0.25% acetic acid and 1.87 μL of methanol per mg of tissue were added to the remaining precipitate (50% methanol extraction). After further homogenization and centrifugation, the newly obtained supernatant was combined with the previously collected supernatant, filtered (Microcon YM‐10), and centrifuged for 90 min at 14000×*g*, and 4°C. The peptide‐containing filtrate was immediately frozen and stored at −80°C until analysis.

## 4 LC–MS/MS Analysis

Peptidome analysis was performed using the nanoAcquity UPLC system coupled to a Synapt G2 mass spectrometer (Waters). Samples (7.5 μL) were loaded onto a Symmetry C18 trapping column (5 μm particles, 180 μm × 20 mm length; Waters) for 5 min at a flow rate of 8 μL/min of phase A (0.1% formic acid) and eluted with a 67 min gradient of 7%–35% of phase B (0.1% formic acid in acetonitrile) through a BEH 130 C18 column (1.7 μm particles, 75 mm × 150 mm, Waters) at a flow of 275 nL/min. Data were acquired using UDMS^E^ data‐independent acquisition mode (Distler et al. 2014) with ion mobility separation (m/z range of 50–2000) in sensitivity mode. Peptide ions fragmentation was achieved via collision induced dissociation (CID), with alternating collision energies (4 eV and 15–65 eV ramp for precursor and fragment ions, respectively). The electrospray ionization (ESI) source was operated in positive mode (3.0 kV capillary voltage, 100°C source temperature, 40 V cone voltage). Glu‐Fibrinopeptide B (500 fmol/mL in 50% MeOH, 0.01% formic acid; Peptide 2.0) was infused through the reference sprayer at 500 nL/min and sampled for 0.5 s every 60 s for mass calibration.

Raw data were processed using Progenesis QI for proteomics (Nonlinear Dynamics, Newcastle, UK). Briefly, a reference run was automatically selected by the software for retention time alignment of all other runs. Normalization was then performed to correct for variations in sample load and ESI signal intensity. This process involves calculating a global scaling factor for each run to equalize the median peptide abundance across all samples, ensuring that valid biological comparisons can be made. Peak picking and normalization were conducted using the software's default parameters. MS/MS spectra were exported as ‘mgf’ files and assessed using PEAKS Studio 7.5 (Bioinformatics Solutions Inc.) for peptide identification. Database search was performed against protein sequences of *Mus musculus* (downloaded from Uniprot, 17.518 entries) using PEAKS DB Search. The parameters used were: 10.0 ppm tolerance for parent mass error tolerance, 0.025 Da for fragments mass, no enzyme, and N‐terminal acetylation (+42.01 Da) and methionine oxidation (+15.99 Da) as variable modifications. Database search results were imported back into Progenesis. For protein‐level quantification, the abundance of each protein was calculated by averaging the signal intensities of its three most intense, unique peptide ions, as previously described (Silva et al. 2006). This method ensures that quantification is based on robust and consistently detected peptides. This analysis compared the normalized abundance of peptide sequences obtained by peptidomics.

## 5 Data Quality Control and Robustness

Given the exploratory nature of this study and the sample size of *n* = 2 animals per group, we performed quantitative quality control analyses to assess the robustness and internal consistency of the peptidomic data. All analyses were performed in R (v4.3.2). Inter‐replicate Correlation: the normalized abundance values for all identified peptides were log2‐transformed. We then calculated the Pearson correlation coefficient between the two biological replicates within each group (WT and KO) for each brain region (total of 4) and extraction method (acidic or alcoholic extraction). Principal component analysis (PCA): to visualize the primary sources of variance in the data, PCA was performed on the log2‐transformed abundance matrix for each dataset using the prcomp function. The scores for the first two principal components were plotted to assess the separation between the WT and KO groups. The results of these analyses (Figure S1, Table S1) demonstrate high within‐group correlation and clear genotype‐driven separation, supporting the reliability of the dataset as a resource.

## 6 Heatmap and GOterm Analysis

This study employs a peptidomic approach, which involves the measurement of the abundance of extracted peptides. We infer changes in the abundance of the parent proteins based on the detection of peptide levels derived from these proteins. This interpretation is based on the assumption that significant, coordinated changes in the levels of multiple peptides from a single protein are likely reflective of changes in the concentration of that parent protein. Therefore, statements regarding “protein abundance” should be interpreted as inferred changes based on the peptidome data. Differentially abundant peptides (DAPs) were defined based on a fold‐change threshold (|Log2FC| > 0.585, corresponding to a > 1.5‐fold change) and *p*_adj < 0.05.

Following preprocessing and normalization, a comprehensive data matrix was constructed incorporating the characteristics of each sample as labels. This compiled data matrix was uploaded to the Morpheus heatmap visualization tool [https://software.broadinstitute.org/morpheus/]. Hierarchical clustering, *Z*‐score normalization, color mapping, and annotations were applied to generate the heatmap, which was used to identify patterns and peptide clusters with consistent abundance changes across brain regions and extraction methods.

Differentially abundant peptides (DAPs) identified by database searching were analyzed using the STRING online database [http://www.string‐db.org/] (Szklarczyk et al. 2019). PPI networks were constructed using an interaction score threshold of > 0.5, indicating a high confidence of interaction. Gene Ontology (GO) enrichment analysis was also conducted using the same interaction score threshold and all identified peptides as the background, focusing on the biological properties of interest. GO analysis encompassed three key aspects: cellular component (CC), molecular function (MF), and biological process (BP), with a minimum of 6 genes required to constitute an enriched GO term. We also employed Metascape, an integrative online resource for functional enrichment, interactome exploration, and gene annotation (Zhou et al. 2019), providing a comprehensive framework for uncovering enriched biological pathways. GraphPad Prism version 7.0 was utilized to generate visual representations of the GO enrichment analysis results and the PPI networks of DAPs.

## 7 Results

**FIGURE 1:**
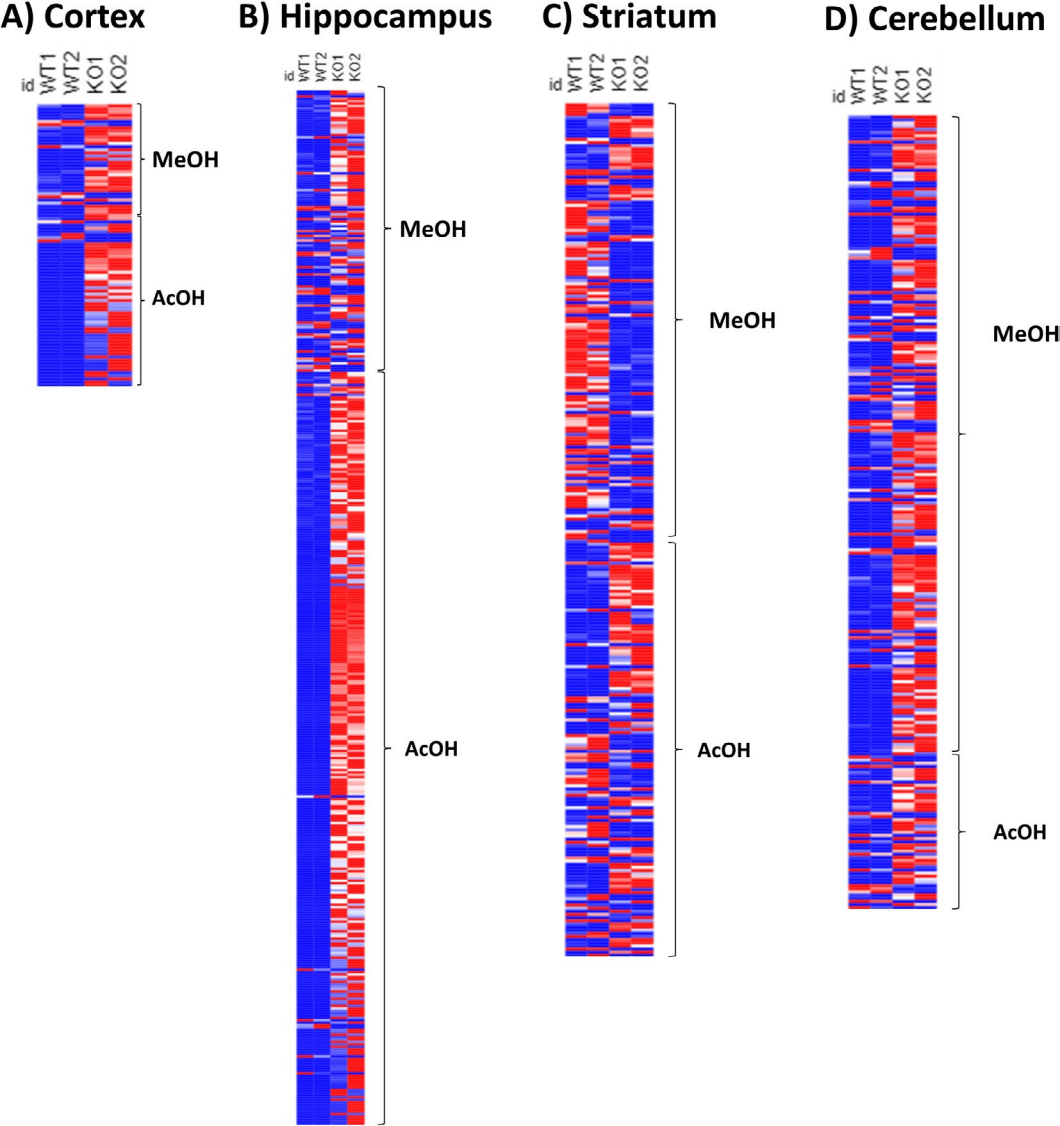
Heatmap illustrating differential peptide abundance in the peptidome of Ndel1 oligopeptidase conditional knockout (Ndel1_cko_) mice compared to mock animals. The brain was dissected into four regions: (A) cortex, (B) hippocampus, (C) striatum, and (D) cerebellum. Peptide abundance was analyzed using two extraction methods: methanol (MeOH) for hydrophobic peptides and acetic acid (AcOH) for hydrophilic peptides. Red represents upregulated peptides, while blue represents downregulated peptides in Ndel1_cko_ compared to mock animals.

Through *in silico* prediction of identified proteins, we detected 397 differentially abundant peptides (DAPs) from alcoholic extraction and 745 DAPs from acidic extraction in the Ndel1 conditional knockout (Ndel1_cko_) mouse peptidome compared to mock controls. The heatmap in Figure 1 demonstrates a predominant upregulation of peptides in Ndel1_cko_ animals across all brain regions (cortex, hippocampus, striatum, and cerebellum) for both acidic (acetic acid) and alcoholic (methanol) extraction methods.

In general, we observed an overall upregulation of peptides across most brain regions, regardless of the specific extraction method employed and with very few discrepancies between the replicates (Figure 1A,B,C), with the notable exception of the striatum (Figure 1C). In this specific brain region, a prominent recovery of DAPs was observed for the methanol extraction, contrasting with the results from the acidic extraction and with other brain regions. Considering methanol might not be highly selective for specific peptides, it is often employed because it is good for broad extraction, while acid conditions are widely used for peptide extraction due to their ability to protonate basic amino acid residues and disrupt ionic interactions, leading to increased peptide solubility and release from complex matrices. The identified peptide sequences used for the analysis presented in this study for each brain region and methods are listed in the Table S2.

**FIGURE 2:**
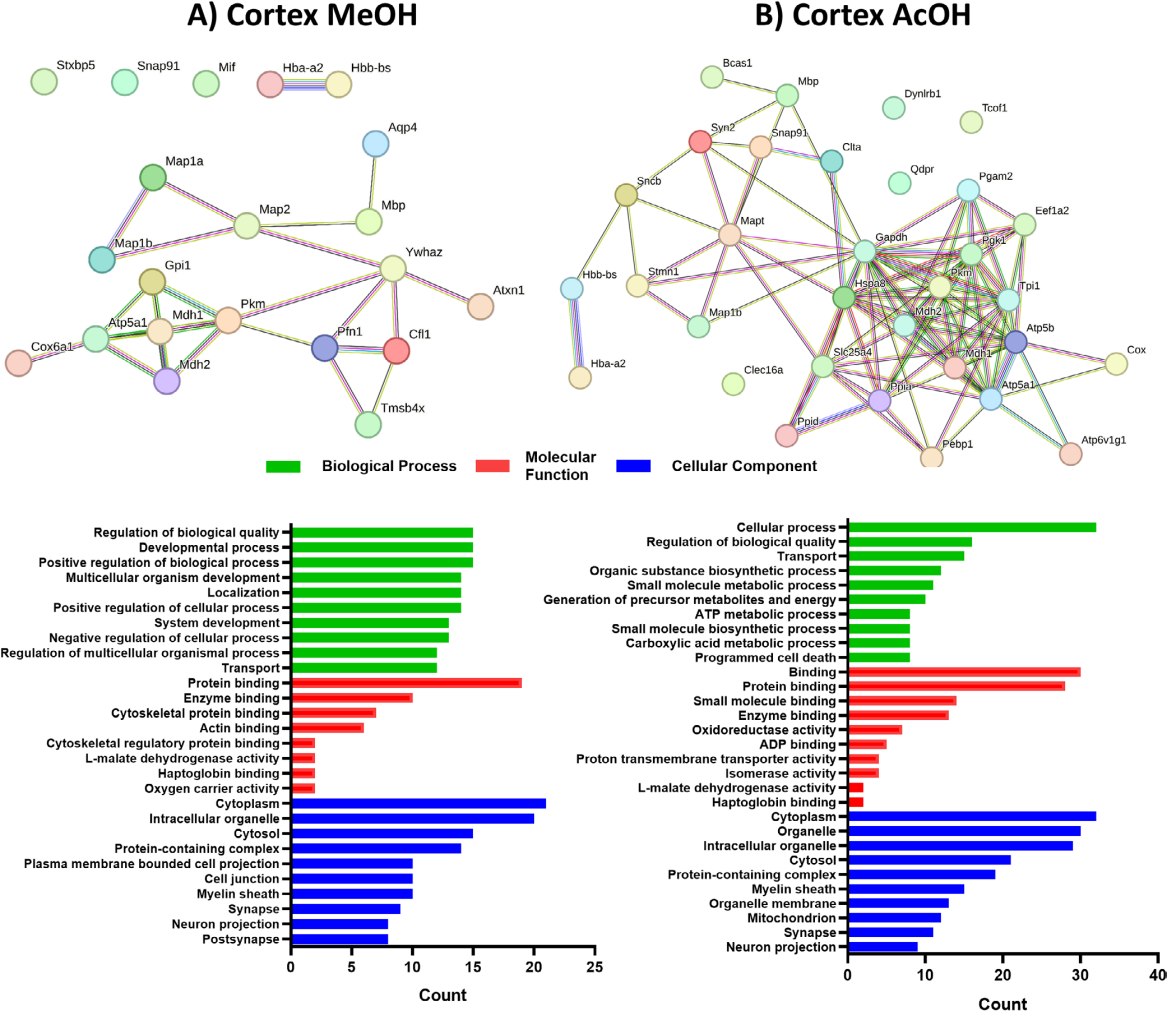
Network interactions and GO enrichment analysis of parent proteins corresponding to the differentially abundant peptides (DAPs) identified in the cortex of Ndel1 oligopeptidase conditional knockout (Ndel1_cko_) mice. (A) Methanol (MeOH) extraction and (B) Acetic acid (AcOH) extraction. Network interactions show relationships among the 39 (MeOH) and 63 (AcOH) unique proteins identified, with significantly differentially abundant proteins inputted into the STRING database [http://www.string‐db.org/] with an interaction score threshold of > 0.5. The nodes represent individual proteins, and the edges represent protein–protein interactions. Below each network, bar plots show the results of Gene Ontology (GO) enrichment analysis for Biological Processes (green), Molecular Functions (red), and Cellular Components (blue).

In the cortex, we identified 39 unique DAPs from alcoholic extraction and 63 from acidic extraction in Ndel1cko compared to mock animals. GO term enrichment analysis categorized these DAPs by biological processes (BPs), molecular functions (MFs), and cellular components (CCs). For alcoholic extraction, BPs were primarily associated with biological quality regulation, developmental processes, and positive regulation of biological processes (38.4% each). MFs were mainly protein binding (48.7%), enzyme binding (25.6%), and cytoskeletal binding (17.9%). For CCs, alcoholic extraction showed enrichment in the cytoplasm (53.8%), intracellular organelles (51.2%), and cytosol (38.4%) (Figure 2A), while acidic extraction showed enrichment in cellular processes (50.7%), regulation of biological quality (25.3%), and transport (23.8%) as the main biological processes. The corresponding MFs included binding (47.6%), protein binding (44.4%), and small molecule binding (22.2%), while the CCs focused on the cytoplasm (50.7%), organelles (47.6%), and intracellular organelles (46%) (Figure 2B).

**FIGURE 3:**
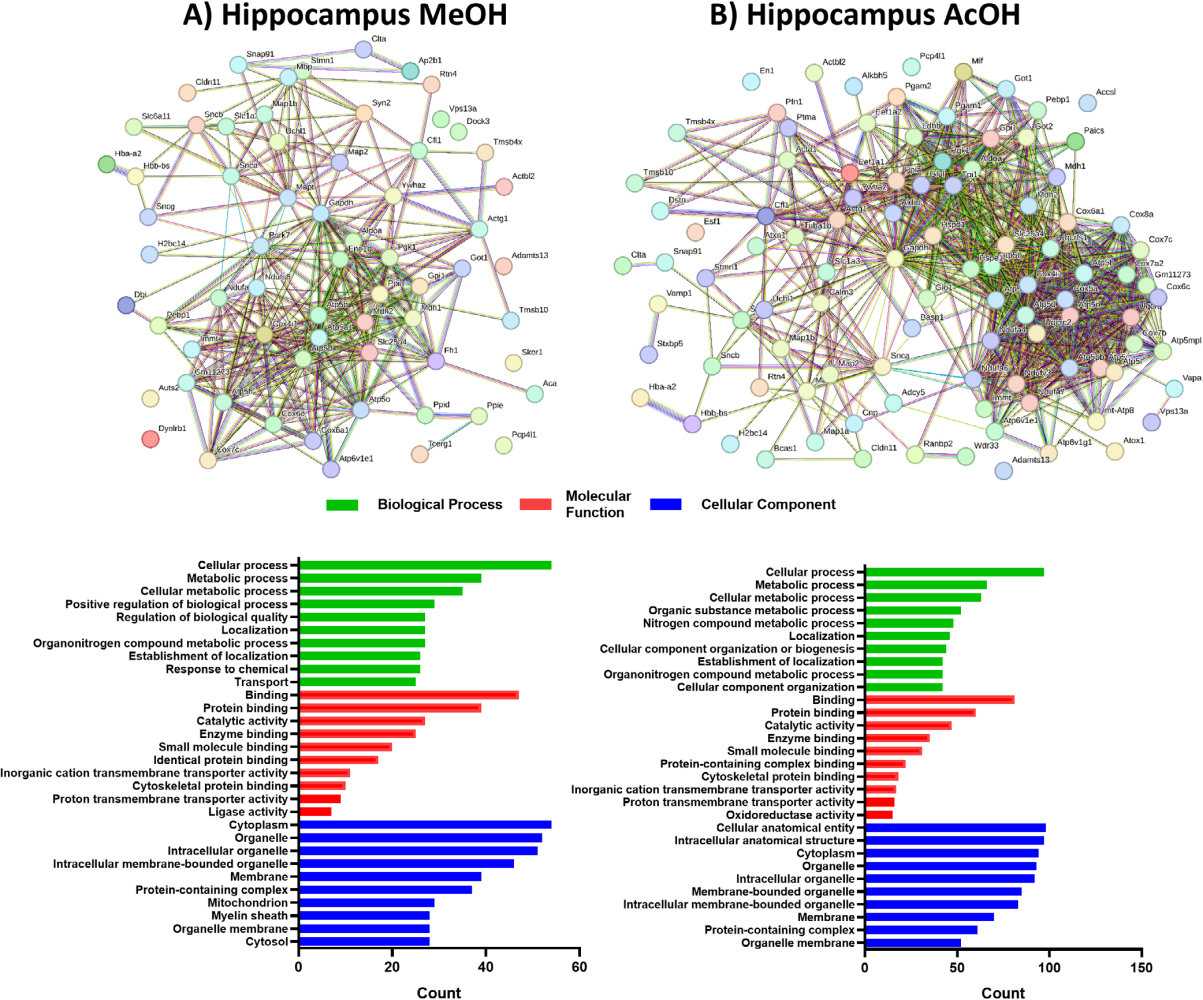
Network interactions depicting the relationships among the 126 and 335 unique proteins identified through alcoholic and acidic extractions, respectively, within the total soluble hippocampus proteome of Ndel1 oligopeptidase conditional knockout mice. (A) Methanol (MeOH) extraction and (B) Acetic acid (AcOH) extraction. The significantly differentially abundant peptides (DAPs) were inputted into the STRING online database (http://www.string‐db.org/), with an interaction score threshold set at > 0.5. This cut‐off point was applied for constructing the protein–protein interaction (PPI) networks of DAPs, as well as for conducting Gene Ontology (GO) enrichment analysis.

Our investigation also revealed a notable difference in the number of identified proteins between Ndel1_cko_ and control mock mice in the hippocampus, with 335 unique proteins detected through alcoholic extraction and 126 through acidic extraction. Notably, both extraction methods primarily yielded GO terms related to BPs. For alcoholic extractions, the main categories were cellular processes (42.8%), metabolic processes (30.9%), and cellular metabolic processes (27.7%), while for acidic extractions these were 28.9%, 19.7%, and 18.8%, respectively. Similarly, the MFs category showed shared dominance in binding (37.9% and 24.1%), protein binding (30.9% and 17.9%), and catalytic activity (21.4% and 14.0%) for alcoholic and acidic extractions, respectively (Figure 3A,B). For CCs, the most enriched GO terms for alcoholic extraction were cytoplasm (42.8%), organelle (41.2%), and intracellular organelle (40.4%) (Figure 3A). In contrast, for acidic extraction, the predominant terms were cellular anatomical entity (29.3%), intracellular anatomical structure (28.9%), and cytoplasm (28.0%) (Figure 3B).

**FIGURE 4:**
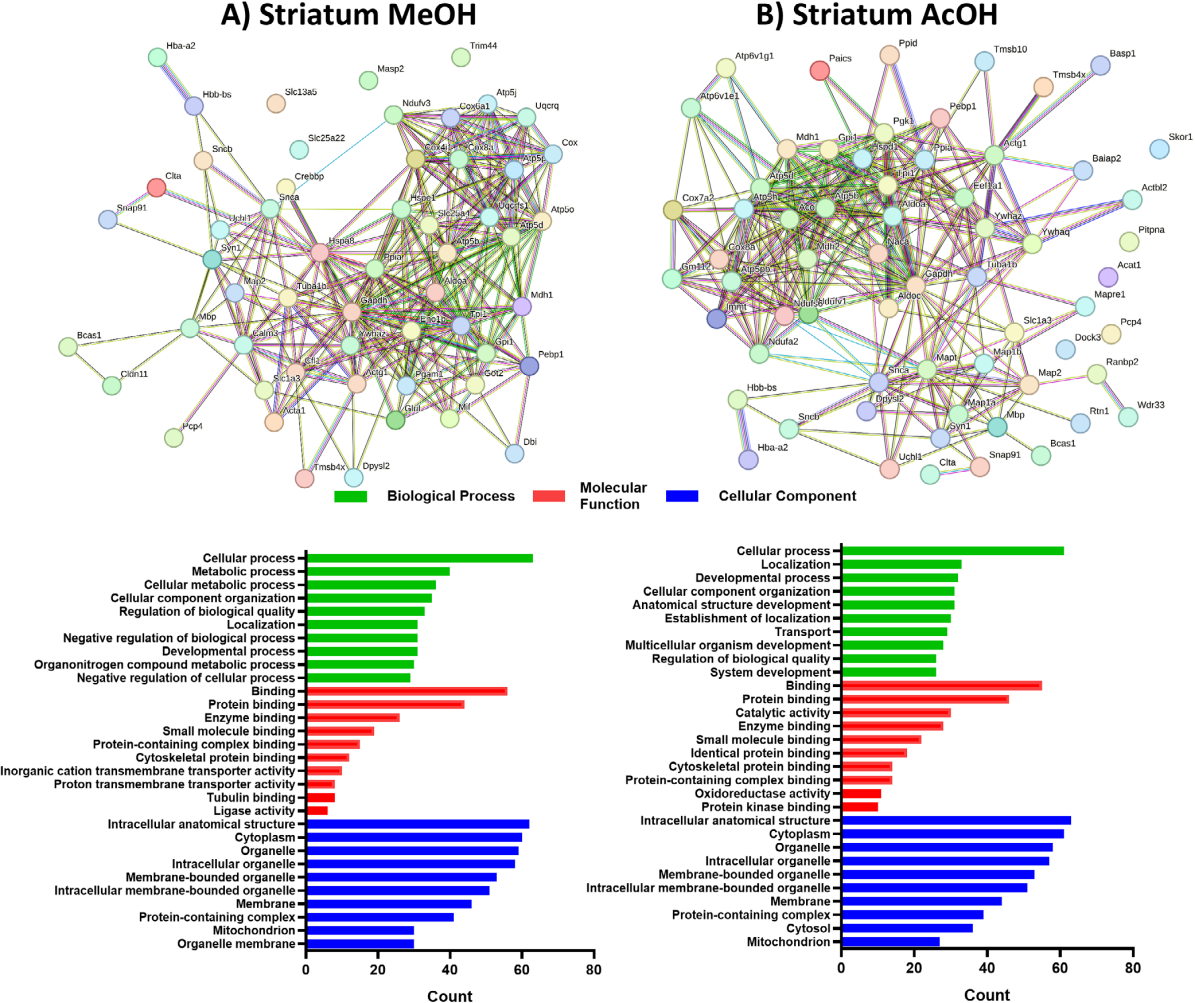
Network interactions depicting the relationships among the 153 and 143 unique proteins identified through alcoholic and acidic extractions, respectively, within the total soluble striatum proteome of Ndel1 oligopeptidase conditional knockout mice. (A) Methanol (MeOH) extraction and (B) Acetic acid (AcOH) extraction. The significantly differentially abundant peptides (DAPs) were inputted into the STRING online database (http://www.string‐db.org/), with an interaction score threshold set at > 0.5. This cut‐off point was applied for constructing the protein–protein interaction (PPI) networks of DAPs, as well as for conducting Gene Ontology (GO) enrichment analysis.

In the striatum, we identified 153 and 143 unique proteins through alcoholic and acidic extractions, respectively, showing that despite the higher recovery noticed for the alcoholic extraction, the protein unique diversity was not significantly different compared to acidic extraction. Notably, the protein regulation shifted from downregulation to upregulation depending on the extraction method used. For alcoholic extraction, GO terms associated with BPs were enriched in cellular processes (41.1%), metabolic processes (26.1%), and cellular metabolic processes (23.5%). The most regulated MFs were binding (36.6%), protein binding (28.7%), and enzyme binding (17.0%). For CCs, the prominent terms were cellular anatomical structure (40.5%), cytoplasm (39.2%), and organelle (38.5%) (Figure 4A). Despite the increased number of upregulated proteins observed for acidic extraction, this method showed similar GO term enrichment patterns to alcoholic extraction. Enriched BPs included cellular processes (42.6%), localization (23.0%), and developmental processes (22.3%). For MFs, the predominant categories were binding (38.4%), protein binding (32.1%), and catalytic activity (20.9%). For CCs, cellular anatomical structure (44.0%), cytoplasm (42.6%), and organelle (40.5%) were the most significant terms (Figure 4B).

**FIGURE 5:**
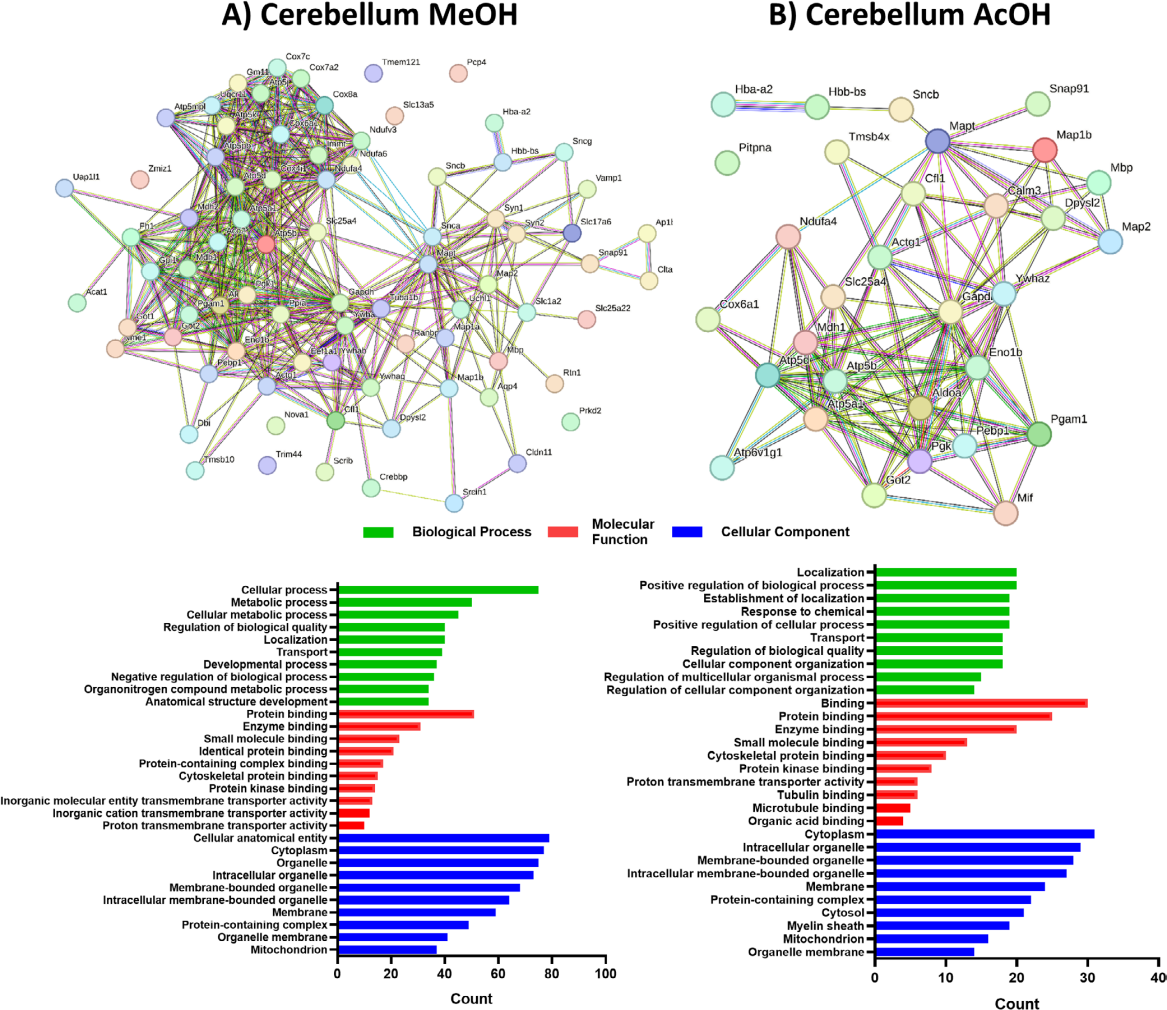
Network interactions depicting the relationships among the 218 and 65 unique proteins identified through alcoholic and acidic extractions, respectively, within the total soluble cerebellum proteome of Ndel1 oligopeptidase conditional knockout mice. (A) Methanol (MeOH) extraction and (B) Acetic acid (AcOH) extraction. The significantly differentially abundant peptides (DAPs) were inputted into the STRING online database (http://www.string‐db.org/), with an interaction score threshold set at > 0.5. This cut‐off point was applied for constructing the protein–protein interaction (PPI) networks of DAPs, as well as for conducting Gene Ontology (GO) enrichment analysis.

In the cerebellum, unlike the striatum but similarly to the hippocampus, the alcoholic extraction yielded a greater number of unique proteins (218 proteins) compared to the acidic extraction (65 proteins). Despite this disparity in protein counts, a consistent trend of downregulated proteins persisted in the Ndel1_cko_ samples. For alcoholic extraction, GO terms associated with regulated proteins in BPs included cellular processes (34.4%), metabolic processes (22.9%), and cellular metabolic processes (20.6%). For MFs, there was predominant regulation in protein binding (23.4%), enzyme binding (14.2%), and small molecule binding (10.5%). For CCs, significant terms included cellular anatomical entity (36.2%), cytoplasm (35.3%), and organelle (34.4%) (Figure 5A). For acidic extraction in the cerebellum, enriched BPs comprised localization (30.7%), positive regulation of cellular processes (30.7%), and establishment of localization (29.2%). For MFs, binding (46.1%), protein binding (38.4%), and enzyme binding (30.7%) were the predominant categories. For CCs, cytoplasm (47.7%), intracellular organelle (44.6%), and membrane‐bounded organelle (43.0%) were the most significant terms (Figure 5B).

**FIGURE 6:**
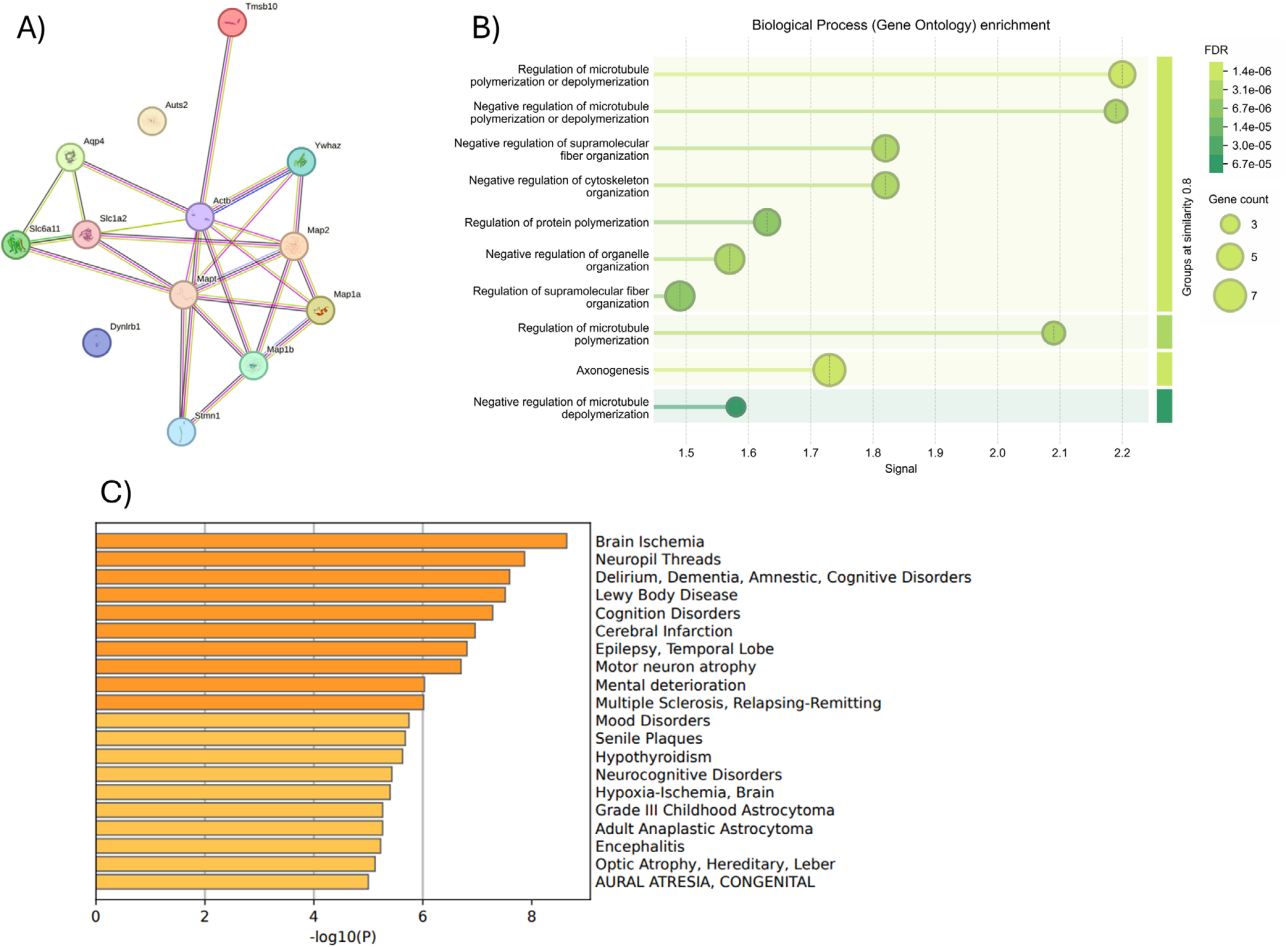
Analysis of protein–protein interaction networks, biological process enrichment, and disease association of differentially abundant peptides (DAPs) in Ndel1_cko_ mice. (A) Protein–protein interaction (PPI) network generated by the STRING platform showing the functional connections between the identified proteins. Lines represent interactions between proteins (nodes). (B) Gene Ontology (GO) biological process enrichment analysis. Bars represent significantly enriched GO terms, with the *X*‐axis indicating the enrichment “Signal”. The size of the circles is proportional to the number of genes in the term, and the color represents the FDR (False Discovery Rate) value, indicating significance. (C) Disease association analysis using the DisGeNET database via Metascape. Orange bars represent diseases significantly associated with the protein set, with the *X*‐axis indicating the −log10(P) of the association.

Moreover, bioinformatic analysis of the differentially expressed peptidome in Ndel1_cko_ mice, using the STRING and Metascape platforms, revealed an intricate network of interactions and biological pathways among the parent proteins of the identified DAPs. In Figure 6A, the protein–protein interaction (PPI) network constructed with STRING demonstrates that the altered proteins, including those crucial for neuronal function and neurotransmission such as *Map1a*, *Map1b*, *Map2*, *Stathmin*, *Actb*, *Ywhaz*, *Mapt*, *Aqp4*, *Slc6a11*, *Slc1a2*, and *Dynll1*, form a functionally connected cluster. This network suggests that alterations in these individual proteins more likely have coordinated and interconnected effects on neuronal biology. GO enrichment analysis (Figure 6B) for biological processes indicates a significant enrichment of pathways associated with cytoskeleton and microtubule organization and dynamics. Finally, Figure 6C displays the disease association analysis, using the DisGeNET database via Metascape. This analysis correlates the set of altered proteins with a variety of neurological and neurodevelopmental conditions. Noteworthy associations include “Brain Ischemia”, “Delirium, Dementia, Amnestic, Cognitive Disorders”, “Lewy Body Disease”, “Cognition Disorders”, “Temporal Lobe Epilepsy,” and “Motor neuron atrophy”. The presence of these associations reinforces the relevance of the identified proteins for neuronal integrity and function, and how their dysregulation may be implicated in mechanisms underlying neurodevelopmental and psychiatric disorders, and synaptic dysfunction. In summary, despite the distinct sets of peptides yielded by each of the two extraction methods employed here, the subsequent pathway analysis consistently pointed to the same core biological processes changed in response to the conditional loss of Ndel1. This convergence strengthens our conclusion that Ndel1 suppression broadly disrupts these specific pathways, such as those related to cytoskeletal organization and proteostasis impairments.

## 8 Discussion

The interactome provides a comprehensive framework for understanding molecular interactions, including protein–protein interactions (PPIs) and the regulation of metabolic networks in specific physiological contexts. PPI analysis is widely used to infer the functions of unknown proteins based on the principle that interacting proteins often share functional similarities and/or common pathways (Cafarelli et al. 2017; Ruas and Guerra‐Sá 2020; Altaf‐Ul‐Amin et al. 2021). These interactions form complex biochemical networks that encompass both functional and physical associations with DNA, RNA, proteins, lipids, and metabolites. Analyzing these networks using proteomic data is essential for elucidating cellular mechanisms and their regulation.

Alcoholic extraction is often employed to analyze compounds with low solubility in water, such as moderately polar and slightly nonpolar compounds. In contrast, acidic extraction increases the solubility of hydrophilic and amphipathic peptides by altering their protonation state as an ionized (charged) compound is more soluble in aqueous solutions. In this study, we utilized two‐step complementary extraction methods, using acetic acid (AcOH) and methanol (MeOH), to assure broad coverage of the peptidome in each brain region. This allowed us to observe possible differences in differentially abundant peptides (DAPs) abundance based on each extraction method for each brain region analyzed here. However, despite variations in the number and direction of change (upregulation/downregulation) of DAPs in each brain region, similar core biological processes and functions were identified by both methods as being affected by the suppression of Ndel1 expression, however with a trend for an increased number of DAPs for MeOH extraction for most brain regions analyzed here. In addition, across all analyzed brains, a significant proportion of DAPs were associated with the upregulation of biological processes related to biological quality regulation, developmental processes, and positive regulation of biological processes, independently of the extraction strategy. In terms of molecular functions (MFs), we observed a broad upregulation associated with protein, enzyme, and cytoskeletal binding. Correspondingly, proteins linked to the cytoplasm, intracellular organelles, and cytosol were among the most frequently identified cellular components (CCs). These observed alterations in biological pathways in Ndel1_cko_ animals align highly well with the numerous reports highlighting Ndel1's established role in intracellular organelle transport via interactions with cytosolic/intracellular cytoskeletal proteins in mature neurons as evaluated mainly in vitro (Shao et al. 2013; Kuijpers et al. 2016; Pandey et al. 2022).

Ndel1 was also demonstrated to be crucial for neurite outgrowth and neuronal migration, processes essential for proper brain formation in vitro and in vivo (Bradshaw and Hayashi 2017; Rodríguez et al. 2020). Disruptions in these processes may contribute to the increased risk of mental disorders, such as schizophrenia (SZ) and autism spectrum disorder (ASD) (Gadelha et al. 2013; Dal Mas, Carvalho, et al. 2019; Nani, Lee, et al. 2020; Courchesne et al. 2024). Building on Ndel1's established role as a cytoskeletal binding protein, we further elucidated its physiological functions using DAP analysis, Gene Ontology (GO) enrichment, PPI networks, and disease association analysis. In fact, we observed a significant upregulation of molecular functions (MFs) related to protein and cytoskeletal binding, consistent with Ndel1's role in regulating microtubule organization and neuronal migration via dynein function (Sasaki et al. 2005; Garrott et al. 2023; Okada et al. 2023). The predominant upregulation of cytoplasmic cellular components (CCs) suggests a compensatory mechanism in response to Ndel1 expression loss, despite its relatively low basal abundance compared to other cytoskeletal proteins such as actin, dynein, and dynactin [https://www.proteinatlas.org/ENSG00000166579‐NDEL1]. On the other hand, proteomic analysis of a DISC1‐overexpressing rat model with suppressed Ndel1 oligopeptidase activity, but with unaffected Ndel1 protein expression, showed mainly synaptic and neurodevelopmental changes, including activation of neuronal and axonal extension processes (Sialana et al. 2018). This supports the hypothesis that Ndel1 protein expression and its oligopeptidase activity may have distinct roles, consistent with our previous findings on Ndel1 oligopeptidase activity in several brain disorders (Gadelha et al. 2013; Dal Mas, Carvalho, et al. 2019; Nani, Lee, et al. 2020; Christoff et al. 2023; Courchesne et al. 2024), as well as in neurite outgrowth process (Kamiya et al. 2006; Hayashi et al. 2010), although further studies, potentially using knock‐in models, are still needed to disentangle the independent effects of oligopeptidase activity and Ndel1 protein structural functions associated with interaction with other proteins. At this point, it is worth emphasizing that Ndel1 interaction with DISC1 protein leads to a competitive inhibition of Ndel1 oligopeptidase activity (Hayashi et al. 2005), further increasing the complexity of evaluating this challenging and complex function of this multifaceted enzyme.

In this study, our peptidome analysis inferred for the first time that several proteins critical for neuronal function and neurotransmission are altered in vivo by evaluating several brain regions of a Ndel1_cko_ mice (Figure 6). Among the most prominently upregulated proteins were those related to cytoskeletal architecture and dynamics, highlighting a compensatory response to cytoskeletal instability, such as the microtubule‐associated proteins MAP1A and MAP1B, as well as MTAP2, which are involved in tubulin polymerization (Rubin and Atweh 2004; Peris et al. 2006; Goodson and Jonasson 2018). Other cytoskeleton‐associated proteins, such as Stathmin, a known regulator of microtubule destabilization, TYB10, involved in cytoskeletal organization, and ACTBL, associated with internal cell motility, were also upregulated (Mader and Brimberg 2019; Cho and Park 2020). This pattern reflects a broad reorganization of the cytoskeleton, consistent with Ndel1's role in microtubule regulation.

Additionally, proteins essential for neuronal physiology and brain homeostasis were elevated. These included AQP4, the most abundant aquaporin in the brain and crucial for water balance and neurovascular coupling, and 14‐3‐3Z, a scaffolding protein involved in signal transduction. We also detected increased levels of proteins implicated in neurodevelopment and neuronal maturation, including TCOF4 (Treacle protein), which plays a role in ribosome biogenesis and neural crest development, and TAU, a well‐known microtubule‐associated protein involved in axonal transport and neurodegeneration (Dixon et al. 2006; Virgilio et al. 2022). These findings are intriguing given that Ndel1 activity in the brain is known to change in response to psychoactive substances (such as amphetamine), and to long‐term treatment with typical or atypical antipsychotics (Bradshaw and Hayashi 2017; Dal Mas, Carvalho, et al. 2019; Nani, Fonseca, et al. 2020; Nani, Lee, et al. 2020; Rodríguez et al. 2020), as well as in the treatment‐resistant SZ patients who usually are medicated mainly with the atypical antipsychotic clozapine (Gadelha et al. 2013). In general, lower Ndel1 activity has been associated with pathological conditions in which impaired neurotransmission would be expected, and this could also indirectly affect the protein expression profile.

Critically, several of the identified DAPs are involved in synaptic function and associated with neurodevelopmental disorders. These included a gene linked to ASD (AUTS2), proteins involved in synaptic vesicle regulation and neurotransmitters transporters, such as SYUA (alpha‐synuclein), and proteins essential for inhibitory and excitatory balance in the CNS, such as S6A11 (GABA transporter 3) and SLC1A2 (glutamate transporter 1). The dynein light chain DYNLRB1, important for intracellular transport and synaptic integrity, was also dysregulated (Pajarillo et al. 2019; Terenzio et al. 2020; Salcedo et al. 2021; Calabresi et al. 2023; Fair et al. 2023). Remarkably, Ndel1 is a well‐known key regulatory protein that interacts directly with the microtubule motor dynein (Zhao et al. 2023; Tsai et al. 2024; Yang et al. 2025).

The maintenance of proteostasis in the brain has been extensively studied within the context of neurodegenerative diseases such as Alzheimer's and Parkinson's diseases, particularly in relation to cellular stress and aging processes. Recent research has proposed that the supersaturation of specific proteins may serve as a key factor in the formation of protein aggregates (Goto et al. 2024). Cellular protein quality control systems operate to remove aberrant and deleterious proteins, thereby preserving cellular proteostasis. More recently, the roles of proteostasis in the context of SZ have also been suggested (Zaharija and Bradshaw 2024; Nucifora et al. 2025). The loss of Ndel1 expression appears to trigger these quality control systems, possibly as a compensatory response to the increased expression of other cytoskeletal proteins. This was observed across most brain regions examined here, with a notable exception for the striatum, which uniquely exhibited a trend towards downregulation of protein expression, which was observed regardless of the employed extraction methods and with an opposite trend to the overall increases in expression of several proteins observed in other brain regions evaluated here.

Functionally, the striatum plays a critical role in coordinating multiple aspects of cognition, including motor planning, decision‐making, motivation, and reinforcement. Dopamine receptors are densely expressed throughout both the dorsal and ventral striatum, and this brain region is intricately involved in synaptic plasticity and motor learning processes (Phillips et al. 2024). Interestingly, decreased Ndel1 activity has been correlated with diminished neurite outgrowth (Hayashi et al. 2010) and altered neuronal positioning in several brain regions, including the striatum and nucleus accumbens (Nani, Fonseca, et al. 2020). Furthermore, Ndel1_cko_ mice, specifically deficient for Ndel1 in forebrain excitatory neurons postnatally, exhibit significant spatial learning and memory deficits, seizures, and a shortened lifespan (Gavrilovici et al. 2021). Notably, the demonstrated spatial learning and memory deficits were associated with deregulation of genes involved in neuronal cell adhesion, plasticity, and neurotransmission, as revealed by genome‐wide transcriptome analysis of the hippocampus of Ndel1_cko_ mice (Kiroski et al. 2020). However, this data needs to be considered with some caution as at P3, the mouse brain's dopamine system is not totally functional and the fundamental hardware of the dopamine system is being established by P3. While individual neurons and synapses are functional, the large‐scale circuits are extremely immature and bear little resemblance to their adult state, which may be advantageous to limit the potential contribution of dopamine signaling modulation for the protein expression evaluated here.

Moreover, deficiency of Ndel1 has also been linked to increased susceptibility to convulsions and epilepsy (Locke et al. 2006; Gavrilovici et al. 2021), and the Ndel1_cko_ mice manifest increased mortality as an epileptic phenotype (Kiroski et al. 2020). Conversely, Ndel1 is upregulated in the hippocampus following status epilepticus (SE), suggesting a potential role for Ndel1 in the pathophysiology of the spontaneous seizure (Wu et al. 2014; Kiroski et al. 2020; Zhu et al. 2020). While one study showed upregulation of Ndel1 expression throughout the hippocampus of adolescent male C57BL/6 mice following pilocarpine‐induced SE (Zhu et al. 2020), another study reported increased Ndel1 levels in the hippocampal blood vessel network but with decreased levels in the CA3 and dentate gyrus regions during the spontaneous seizure period post‐SE in C57BL/6 mice (Wu et al. 2014). Furthermore, the interaction between Ndel1 and DISC1 has been proposed to be protective through ERK signaling during the spontaneous seizure period after pilocarpine‐induced SE (Wu et al. 2014). Our findings align with this literature, as the hippocampus was the brain region with the highest number of DAPs, and disease association analysis pointed to epilepsy, reinforcing Ndel1's crucial role in this condition, as suggested by others (Choi et al. 2016; Zhu et al. 2020; Gavrilovici et al. 2021; Tsai et al. 2024).

In this study, the complete suppression of Ndel1 protein expression in mice reinforced its critical role in maintaining cytoskeletal protein homeostasis. Notably, we found no direct evidence of Ndel1 involvement in dopamine and serotonin signaling pathways. However, we identified DAPs related to GABAergic and glutamatergic pathways, indicating potential neurotransmission imbalances in Ndel1_cko_ animals. It remains plausible that Ndel1's impact on neuronal signaling, as observed in patients and rodent models, may be modulated by Ndel1 oligopeptidase activity levels or by the putative metabolites generated by this enzyme. Therefore, a future aim is the analysis and identification of Ndel1's endogenous peptide substrates to directly assess the products of the oligopeptidase activity of Ndel1. Our study effectively demonstrates the viability of employing proteomic analysis based on peptidome data, with future improvements aimed at identifying Ndel1's natural substrates and its key metabolites, supporting the validity of the concomitant use of alcoholic and acid extractions.

Previous studies on prolyl oligopeptidase (POP), another oligopeptidase linked to proteostasis‐related disorders (Hannula et al. 2013; Svarcbahs et al. 2019; Walczewska‐Szewc et al. 2022; Eteläinen et al. 2023), have shown that POP deficiency in mice disrupts synaptic plasticity, reduces anxiety‐like behavior, and decreases body weight and brain volume (Höfling et al. 2016). These effects resemble those also observed in Ndel1_cko_ mice (Kiroski et al. 2020). However, it was also demonstrated that the ability of POP inhibitors to influence brain proteostasis cannot be predicted solely by their inhibitory efficacy (Kilpeläinen et al. 2020), despite their success in treating conditions like acute respiratory distress syndrome (Zerikiotis et al. 2023), pulmonary fibrosis (Cucinotta et al. 2023), and tauopathy (Eteläinen et al. 2023) in animal models. Although POP knockout models have shown potential in mitigating alpha‐synuclein toxicity—manifested as reduced locomotor activity and lower striatal dopamine levels (Svarcbahs et al. 2019)—the exact effects of POP inhibitors on brain proteostasis remain unclear. For instance, a 4‐day POP inhibition regimen produced significant changes in the rat brain peptidome, yet the peptides identified did not align with the known proline‐specific cleavage preference of POP (Tenorio‐Laranga et al. 2012). This apparent discrepancy between POP knockout and inhibitor‐based studies may be explained by the impact of POP inhibitors on the enzyme's conformational flexibility. This concept is also supported by studies on POP, where defects in neuronal growth cone formation in POP‐negative mice were rescued by a catalytically inactive POP mutant, providing direct evidence that the protein's structural role and interactions can be more critical than its enzymatic activity in certain developmental processes (Di Daniel et al. 2009). POP specific inhibitors may also alter its dynamics, reducing POP's ability to bind or interact with other proteins, which is a key factor in maintaining proteostasis (Savolainen et al. 2015; López et al. 2016). Similar mechanisms may also potentially apply to Ndel1 (Hayashi et al. 2005; Bradshaw and Hayashi 2017), discouraging us from focusing on Ndel1 inhibitors, although further studies are still needed to clarify this specific point.

Thus, we believe that using a conditional knockout mouse model to assess protein expression represents several advantages, which in addition to the approach evaluating a model with decreased Ndel1 activity without interfering in Ndel1 protein expression (Sialana et al. 2018; Nani, Fonseca, et al. 2020), could help clarify Ndel1's primary in vivo function. A significant limitation of our approach, however, was the high rate of lethality observed in the knockout animals, even with the conditional strategy that we may upgrade to a more controlled model in the near future. Nevertheless, the analysis of each brain region separately allowed increasing the confidence of our conclusions, as the same pathways were found enriched here regardless of the extraction method or the specific brain region analyzed. In essence, by inferring which proteins are affected by the loss of Ndel1, we have reinforced its critical role in maintaining cytosolic protein homeostasis, with a significant enrichment of pathways associated with cytoskeleton organization and dynamics. The dysregulation of these pathways correlates with a variety of neurological and neurodevelopmental conditions, reinforcing the relevance of the identified proteins for neuronal integrity and function (Youn et al. 2009; Hayashi et al. 2010; Hippenmeyer et al. 2010; Shao et al. 2013; Jiang et al. 2016; Kuijpers et al. 2016; Bradshaw and Hayashi 2017; Garrott et al. 2022, 2023; Tsai et al. 2024). Due to the wide range of biological processes and disease associations identified here, confirming how each dysregulation contributes to the mechanisms of neurodevelopmental disorders could not be addressed in detail in the present work. Therefore, our next goal involving a peptidome analysis focused on the identification of potential native substrates of Ndel1 will be performed with an improved model of Ndel1_cko_ mice, aiming to ultimately dissociate the effects of its hydrolytic activity from its protein–protein interaction capabilities (Hayashi et al. 2005, 2010), as well as assuring a higher survival rate of these animals.

A key consideration in interpreting these results is that the loss of the Ndel1 protein precludes the differentiation between its enzymatic functions as an oligopeptidase and its structural roles as a scaffolding protein. In addition, interaction with some proteins, such as DISC1, also determines oligopeptidase activity suppression. The widespread changes we observe in peptides derived from cytoskeletal and synaptic proteins could arise from either the loss of direct peptide processing by Ndel1 or from broader, indirect disruptions to microtubule dynamics and intracellular transport. Future studies employing knock‐in mice expressing a catalytically‐dead Ndel1 mutation would contribute to dissecting which of the peptidomic changes observed here are direct consequences of lost enzymatic activity versus disruptions to its scaffolding functions.

A notable limitation of this study is the sample size of animals per group, which precludes robust statistical inference and was necessitated by the high rate of lethality observed in the *Ndel1*_cKO_ model. In addition, our data reveal distinct peptidomic signatures across the four brain regions analyzed, with the striatum showing a particularly unique profile. This regional heterogeneity could stem from several factors. First, the efficiency of mosaic CMV‐Cre recombination may vary between regions. Second, and perhaps more biologically significant, Ndel1 may have distinct functional roles within the unique cellular architecture of the striatum compared to the hippocampus and cortex. Furthermore, as this study utilized bulk brain tissue from a mosaic knockout model, the observed peptidome reflects a composite of changes from all cell types. In other words, one needs to consider that the reproducibility and robustness of the present analysis could also be drawn by the consistent similar pathways identified for all brain regions analyzed, regardless of increased or decreased DAP, reinforcing that Ndel1 suppression affected the same core biology, mainly associated with neuronal integrity and function.

These findings may help elucidate the molecular mechanisms underlying mental disorders development and other neurodevelopmental illnesses. Our methodology, as validated here, enables the identification of differentially expressed proteins by analyzing the peptidome of several brain regions of this specific conditional knockout animal model (i.e., Ndel1_cko_ mice), underscoring its association with diverse pathologies, including brain ischemia, delirium, dementia, amnestic cognitive disorders, Lewy body disease, temporal lobe epilepsy, and motor neuron atrophy, but not necessarily with psychiatric conditions. The efficacy of alcoholic and acidic extractions for identifying differentially expressed proteins in brain was also reinforced. A deeper understanding of the pathways modulated by this potential biomarker of neurodevelopment and pathological conditions of mental illness may help to pave the way for novel therapies needed for multiple debilitating brain disorders, also highlighting the Ndel1 roles in epilepsy. Ultimately, this research may contribute to a deeper understanding of Ndel1's role not only in brain development, but also in its neural plasticity and function at early developmental phase, clarifying how this enzyme could contribute to the etiology of psychiatric and other brain disorders.

## Author Contributions

**João V. Nani:** methodology, software, formal analysis, data curation, validation, visualization, writing – review and editing, writing – original draft. **Jackelinne Yuka Hayashi:** methodology, investigation, validation, visualization, writing – review and editing, formal analysis. **Joana D. Campeiro:** methodology, investigation, validation, visualization, writing – review and editing. **Guilherme Araújo Câmara:** methodology, software, formal analysis. **Atsushi Saito:** methodology, conceptualization, investigation, writing – review and editing. **William Y. Oyadomari:** writing – review and editing. **Akira Sawa:** investigation, methodology, validation, visualization. **Atsushi Kamiya:** methodology, writing – review and editing, investigation. **Alexandre K. Tashima:** methodology, writing – review and editing, writing – original draft, data curation, supervision, formal analysis, validation, visualization, investigation. **Mirian A. F. Hayashi:** conceptualization, investigation, funding acquisition, writing – original draft, writing – review and editing, visualization, project administration, resources, supervision.

## Funding

This work was supported by Coordenação de Aperfeiçoamento de Pessoal de Nível Superior, Financial Code 001. Conselho Nacional de Desenvolvimento Científico e Tecnológico, 310057/2023‐0, 39337/2016‐0. Financiadora de Estudos e Projetos, 04.16.0054.02. Fundação de Amparo à Pesquisa do Estado de São Paulo, 2011/50963‐4, 2014/50891‐1, 2017/02413‐1, 2019/08287‐3, 2019/09207‐3, 2019/13112‐8, 2020/01107‐7, 2022/00527‐8, 2022/03297‐3, 2023/07904‐4. Atsushi Kamiya: MH136297 and DA060630.

## Conflicts of Interest

The authors declare no conflicts of interest.

## Supporting information


**Figure S1:** Quality control and distribution analysis of peptidomic data. (A) Principal component analysis (PCA) for each brain region and extraction method. The score plots show the separation of samples along the first two principal components. In all conditions, principal component 1 (PC1), which accounts for the largest source of variance, clearly separates the samples by genotype (WT, blue vs. KO, red). This demonstrates high consistency between biological replicates (*n* = 2) and indicates that the *Ndel1* deletion is the primary driver of variation in the data. (B) Distribution of Log2 Fold Changes for all quantified peptides. The histograms display the distribution of peptides based on their log2 fold change (Log2(KO/WT)) for each brain region and extraction method (Acetic Acid, red; Methanol, blue). Most peptides cluster around zero (dashed line), indicating no change, while the tails of the distribution represent the differentially abundant peptides.
**Table S1:** Correlation analysis between replicates for each condition and brain region.

**Table S2:** List of identified peptide sequences used for the analysis presented in this study for each brain region and extraction methods. This table includes the comprehensive peptidomic quantification datasets for Ndel1 conditional knockout and control mice.

## Data Availability

The peptidomic quantification datasets generated and analyzed for this study are publicly available in the Zenodo repository. This includes the comparative analysis spreadsheets for all four brain regions (cortex, hippocampus, striatum, and cerebellum) under both acidic and alcoholic extraction methods. The data can be accessed via the following identifier: http://doi.org/10.5281/zenodo.17535597.
