# Supplementary material for: Postnatal Mouse Brain Region‐Resolved Peptidomic Resource of Conditional Ndel1 Loss: Comparison of Acidic and Alcoholic Extraction Strategies
Source: J Neurochem. 2025 Dec 23;169(12):e70330. doi: 10.1111/jnc.70330 (PMC12724395; doi:10.1111/jnc.70330)
Supplement: Supplementary file 1 — Figure S1: Quality control and distribution analysis of peptidomic data. (A) Principal component analysis (PCA) for each brain region and extraction method. The score plots show the separation of samples along the first two principal components. In all conditions, principal component 1 (PC1), which accounts for the largest source of variance, clearly separates the samples by genotype (WT, blue vs. KO, red). This demonstrates high consistency between biological replicates (n = 2) and indicates that the Ndel1 deletion is the primary driver of variation in the data. (B) Distribution of Log2 Fold Changes for all quantified peptides. The histograms display the distribution of peptides based on their log2 fold change (Log2(KO/WT)) for each brain region and extraction method (Acetic Acid, red; Methanol, blue). Most peptides cluster around zero (dashed line), indicating no change, while the tails of the distribution represent the differentially abundant peptides. Table S1: Correlation analysis between replicates for each condition and brain region. [file JNC-169-0-s001.pdf]

# A Region-Resolved Peptidomic Resource of Conditional Ndel1 Loss in the Postnatal Mouse Brain

João V. Nani[1,#,*], Jackelinne Yuka Hayashi[2,#], Joana D. Campeiro[1,#], Guilherme Araújo Câmara[2], Atsushi Saito[3], William Y. Oyadomari[1], Akira Sawa[3,4,5,6,7,8], Atsushi Kamiya[3,4], Alexandre Tashima[2], Mirian A. F. Hayashi[1,*]

[1]Department of Pharmacology, Escola Paulista de Medicina (EPM), Universidade Federal de São Paulo (UNIFESP), SP, Brazil; [2]Department of Biochemistry , Escola Paulista de Medicina (EPM), Universidade Federal de São Paulo (UNIFESP), SP, Brazil; [3]Department of Psychiatry and Behavioral Sciences, [4]Department of Neuroscience, [5]Department of Biomedical Engineering, [6]Department of Genetic Medicine, [7]Department of Pharmacology, Johns Hopkins University School of Medicine, Baltimore, MD, USA; [8]Department of Mental Health, Johns Hopkins University Bloomberg School of Public Health, Baltimore, MD, USA.

*Corresponding author:

Prof. Mirian A. F. Hayashi, *Ph.D.*

e-mail: mhayashi@unifesp.br or mirianhayashi@yahoo.com

and

João Nani, *Ph.D.*

e-mail: joao.nani@unifesp.br

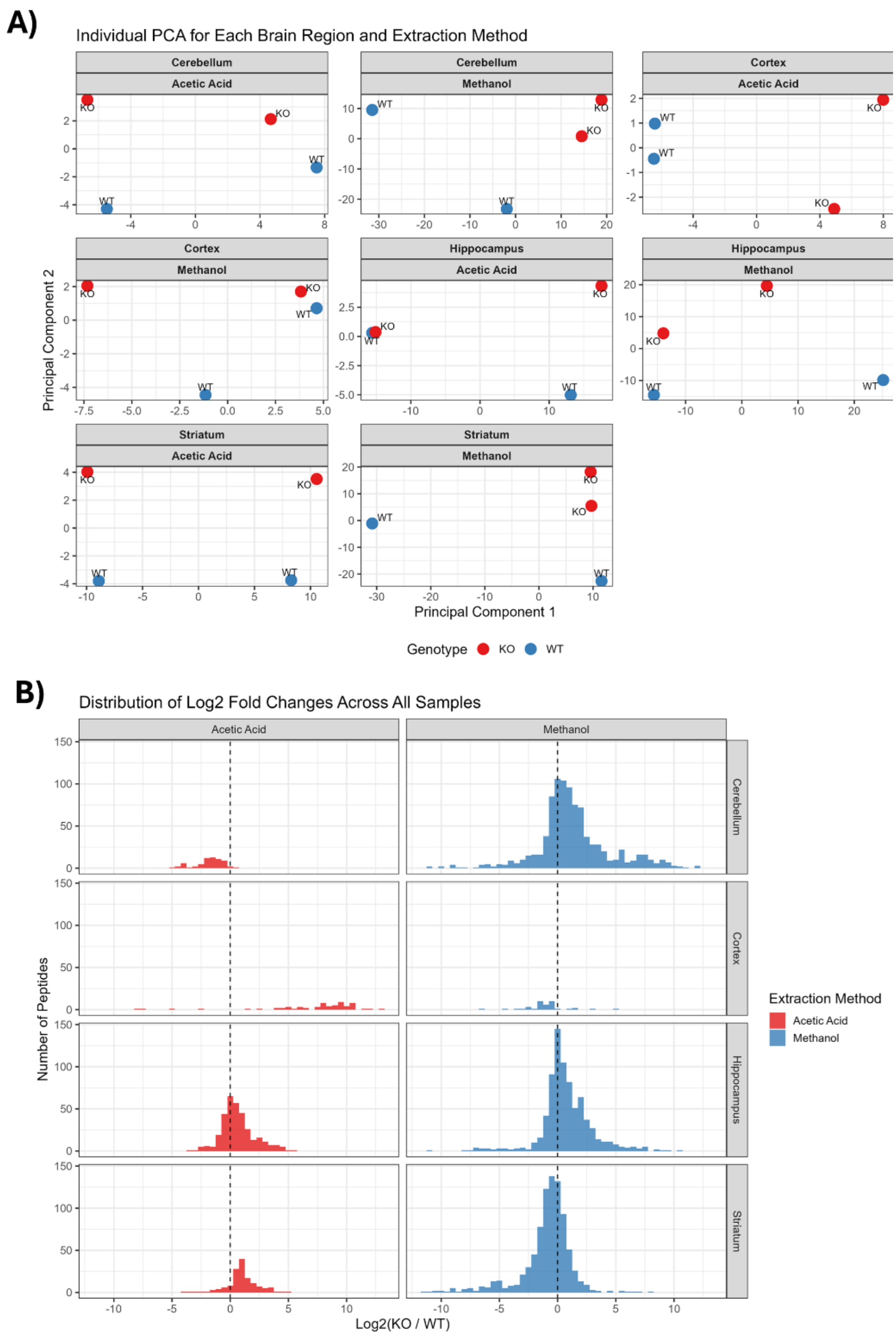


**Supplementary Figure 1: Quality Control and Distribution Analysis of Peptidomic Data. (A)** Principal Component Analysis (PCA) for each brain region and extraction method. The score plots show the separation of samples along the first two principal components. In all conditions, Principal Component 1 (PC1), which accounts for the largest source of variance, clearly separates the samples by genotype (WT, blue vs. KO, red). This demonstrates high consistency between biological replicates (n=2) and indicates that the *Ndel1* deletion is the primary driver of variation in the data. **(B)** Distribution of Log2 Fold Changes for all quantified peptides. The histograms display the distribution of peptides based on their log2 fold change (Log2(KO/WT)) for each brain region and extraction method (Acetic Acid, red; Methanol, blue). Most peptides cluster around zero (dashed line), indicating no change, while the tails of the distribution represent the differentially abundant peptides.

Supplementary Table 1. Correlation analysis between replicates for each condition and brain region

| Brain Region | Extraction Method | WT Replicate Correlation (r) | KO Replicate Correlation (r) |
|---|---|---|---|
| Cerebellum | Acetic Acid | 0.220 | 0.450 |
| Cerebellum | Methanol | 0.713 | 0.804 |
| Cortex | Acetic Acid | 0.854 | 0.639 |
| Cortex | Methanol | 0.601 | 0.465 |
| Striatum | Acetic Acid | 0.545 | 0.398 |
| Striatum | Methanol | 0.536 | 0.902 |
| Hippocampus | Acetic Acid | 0.511 | 0.460 |
| Hippocampus | Methanol | 0.699 | 0.640 |
